# Biomarker profiling for risk of future heart failure (HFpEF) development

**DOI:** 10.1186/s12967-021-02735-3

**Published:** 2021-02-09

**Authors:** Chris J. Watson, Joe Gallagher, Mark Wilkinson, Adam Russell-Hallinan, Isaac Tea, Stephanie James, James O’Reilly, Eoin O’Connell, Shuaiwei Zhou, Mark Ledwidge, Ken McDonald

**Affiliations:** 1 Wellcome-Wolfson Institute for Experimental Medicine, Queen’s University, Belfast, BT9 7BL Northern Ireland; 2 Conway Institute, University College Dublin, Dublin 4, Ireland; 3 St. Vincent’s University Hospital Healthcare Group, Dublin 4, Ireland; 4 Internal Medicine, Lankenau Medical Center, Wynnewood, PA 19096 USA

**Keywords:** Heart failure, HFpEF, Risk factors, Biomarkers

## Abstract

**Background:**

The purpose of this study was to investigate the utility of BNP, hsTroponin-I, interleukin-6, sST2, and galectin-3 in predicting the future development of new onset heart failure with preserved ejection fraction (HFpEF) in asymptomatic patients at-risk for HF.

**Methods:**

This is a retrospective analysis of the longitudinal STOP-HF study of thirty patients who developed HFpEF matched to a cohort that did not develop HFpEF (n = 60) over a similar time period. Biomarker candidates were quantified at two time points prior to initial HFpEF diagnosis.

**Results:**

HsTroponin-I and BNP at baseline and follow-up were statistically significant predictors of future new onset HFpEF, as was galectin-3 at follow-up and concentration change over time. Interleukin-6 and sST2 were not predictive of future development of new onset HFpEF in this study. Unadjusted biomarker combinations of hsTroponin-I, BNP, and galectin-3 could significantly predict future HFpEF using both baseline (AUC 0.82 [0.73,0.92]) and follow-up data (AUC 0.86 [0.79,0.94]). A relative-risk matrix was developed to categorize the relative-risk of new onset of HFpEF based on biomarker threshold levels.

**Conclusion:**

We provided evidence for the utility of BNP, hsTroponin-I, and Galectin-3 in the prediction of future HFpEF in asymptomatic event-free populations with cardiovascular disease risk factors.

## Background

The epidemiological horizon for heart failure remains a major public health concern. While significant pharmacological and device-based advances have been made in heart failure with reduced ejection fraction (HFrEF), successful strategies for preserved ejection fraction (HFpEF) remain elusive. Recent observations of the growing prevalence of this phenotype heightens concerns. Furthermore, studies including the St Vincent’s Screening to Prevent Heart Failure (STOP-HF) study have reported higher rates of asymptomatic left ventricular diastolic rather than systolic dysfunction in a large community cohort at-risk of heart failure [1, 2]. These facts underline the need to develop a focused preventative approach for HFpEF, a strategy that requires clear definition of those at highest risk of progression.

Development of significant asymptomatic left ventricular diastolic dysfunction (LVDD) has been shown to be an independent predictor of later development of HFpEF [3–5]. Progression of LVDD, shown to occur among 13–28% of these cohorts, is also associated with development of heart failure. However, as the pathophysiology of HFpEF in increasingly understood to be driven by fibro-inflammation and cardiomyocyte damage, factors other than asymptomatic LVDD predict later development of HFpEF and indeed many new cases of HFpEF develop without interval change in LVDD or other structural or functional abnormalities of the heart [6].

Taken together, these observations support biomarker profiling to enhance and refine risk prediction. In particular, point-in-time assessment of natriuretic peptide, Galectin-3, and high sensitive (hs) Troponin levels, all reflect background pathological change linked to new onset HFpEF and have been shown either on their own, or as part of a multi-marker approach, to independently predict new onset HF [7–12]. In addition, markers of inflammatory processes linked to the pathophysiology of HFpEF may pose as biomarker candidates, including interleukin-6 (IL-6) and soluble suppression of tumorigenicity 2 (sST2) [6, 13–17]. Analysis of the change value of these circulating proteins over time in at-risk populations may add to the clinical value of this biomarker approach as longitudinal changes may reflect ongoing change in subclinical pathology, thereby improving our ability to predict and track risk in at–risk cohorts.

Therefore in this study, we first analyzed the value of baseline and interval change values of biomarkers associated with fibro-inflammation to identify associates of new-onset HFpEF within the STOP-HF population (measurements taken during asymptomatic period). Secondly, we assessed whether sequential change in combinations of biomarker profiles can improve risk stratification in this group, thereby allowing for better determination of those at risk for the later development of HFpEF. Finally, we derived a clinical prediction rule based on biomarker combinations.

## Methods

### Study population

The study population consisted of 90 patients selected from within the longitudinal STOP-HF study, which comprises asymptomatic patients with risk factors for the future development of heart failure and has been described before [1]. All study participants gave written informed consent to join the study. The study protocol was approved by the ethics committee of St. Vincent's University Hospital, Dublin, which conformed to the principles of the Helsinki Declaration. Thirty of these patients developed HFpEF over time, and this cohort were age and sex matched 2:1 to a cohort that did not develop HFpEF (n = 60) over a similar time period. A panel of 5 disease associated biomarkers were quantified in all patients at two time points prior to the development of HFpEF.

HFpEF diagnosis was confirmed as new onset symptoms consistent with heart failure, presenting in the community or to hospital, with proven left ventricular ejection fraction (LVEF) > 50% an elevated BNP (> 100 pg/ml,) in the presence of Doppler-echocardiographic evidence of diastolic dysfunction of the left ventricle (LV) in line with European Society of Cardiology guidelines.

### Biomarker profiling

Peripheral venous blood samples were collected during all clinical visits as part of the STOP-HF programme and serum samples were generated and stored at -80 °C for future use as described previously [18]. During these clinical visits B-type natriuretic peptide (BNP) was measured (Triage, Biosite). Additional biomarker analysis was performed on baseline and follow up samples to quantify levels of Galectin-3 and hsTroponin I (Architect System, Abbott), sST2 (Presage ST2 Assay, Critical Diagnostics), and IL-6 (electro-chemiluminescence assay, Mesoscale Discovery).

### Clinical relative risk matrix

Relative risk models of new onset HFpEF were developed stratified by biomarker thresholds of those with significant associations to the future development of HFpEF. First, we grouped patients into groups of approximate quintiles of BNP. Within each quintile we selected sub sub-categories of patients based on whether they had above or below median levels of other biomarkers in order of the strength of association of biomarkers with new onset HFpEF in the multivariable analysis. Within the resulting sub-categories of patient, we calculated the relative risk of the development of HFpEF over the follow up period using the lowest sub-category as the reference (unity). In presenting the table of probabilities by biomarker thresholds, we used the following convention to categorise the risk probability: < 10 low risk; 10–20 intermediate risk; > 20 high risk.

### Statistical analysis

Summary statistics for continuous variables are presented as median [inter-quartile range]—50th [25th:75th] percentiles—and for binary variables as n (%). Statistical tests for differences on continuous variables between the HFpEF progressor and non progressor cohorts were Student t-test or Wilcoxon signed-rank test, depending on whether the variables were normally distributed. The Shapiro–Wilk test was used to test whether variables could be considered normally distributed with alpha set at 0.05. Statistical tests for differences on binary variables between two cohorts were the Chi-squared test or Fisher’s exact test, depending on whether the n in any cell in the 2 × 2 summary table was less than 5. Unadjusted and adjusted logistic regression models were employed to quantify and test the relationships of biomarkers as well as echocardiography parameters with the HFpEF progression prevalence. Regression models were adjusted for all other biomarkers at the corresponding time-point. Results were presented in the form of the odds ratio and area under curve (AUC) with 95% confidence intervals. A Repeated-Measures ANOVA analysis was applied to determine if there was a significant difference in biomarker levels between study time points. Unadjusted and adjusted (age/sex) Cox proportional hazards analysis was used to quantify and test the relationships between the biomarker variables and the outcome of time to HFpEF diagnosis, presented in the form of hazard ratios.

## Results

### Baseline characteristics

The baseline features of the study population are highlighted in Table 1. The patient population consisted of 30 patients with new onset HFpEF over time (HFpEF progressor) age and sex matched with 60 patients who did not develop HFpEF over a similar time period (HFpEF non-progressor). Medication use in both cohorts was similar. Baseline echocardiographic differences between the study groups include expected higher left atrial volume index (LAVI) and left ventricular mass index (LVMI) in the HFpEF progressor cohort.

**Table 1:** Baseline patient characteristics of HFpEF progressor and non-progressor cohorts

Median [IQR] or N (%)	Total study cohort	HFpEF progressor	HFpEF Non progressor	*p*-value
N	90	30	60	
Age	75.0 [69.1:78.2]	76.0 [69.8:78.1]	74.3 [68.8:78.2]	0.4828
Male	42 (46.7%)	14 (46.7%)	28 (46.7%)	> 0.99
BMI	29.0 [25.0:34.5]	33.0 [25.2:36.8]	27.0 [25.0:30.0]	0.0710
SBP	137.5 [124.2:151.0]	128.0 [111.2:144.5]	140.5 [130.0:151.5]	0.1181
DBP	78.0 [70.2:84.0]	73.5 [65.2:82.5]	80.0 [75.8:86.0]	0.0197
HDL	1.2 [1.0:1.5]	1.2 [1.0:1.5]	1.2 [1.0:1.5]	0.7979
Triglycerides	1.6 [1.1:2.0]	1.6 [1.1:2.0]	1.5 [1.1:2.1]	0.8742
RAAS	69 (76.7%)	24 (80.0%)	45 (75.0%)	0.7920
AA	0 (0.0%)	0 (0.0%)	0 (0.0%)	-
ARB	38 (42.2%)	14 (46.7%)	24 (40.0%)	0.7060
ACEI	35 (38.9%)	13 (43.3%)	22 (36.7%)	0.7020
Diuretic	40 (44.4%)	18 (60.0%)	22 (36.7%)	0.0610
BB	48 (53.3%)	19 (63.3%)	29 (48.3%)	0.2620
CCB	45 (50.0%)	17 (56.7%)	28 (46.7%)	0.5020
Anti-platelet	59 (65.6%)	22 (73.3%)	37 (61.7%)	0.3880
EF	67.0 [62.0:72.8]	66.0 [62.0:72.0]	68.0 [62.0:73.0]	0.4690
Lateral e’	7.1 [5.8:8.8]	7.8 [6.2:10.5]	6.5 [5.5:8.2]	0.0298
Peak E	73.0 [62.0:86.0]	79.5 [69.8:95.0]	69.0 [58.0:81.0]	0.0016
E / lat. e’	10.4 [8.0:13.7]	10.4 [8.2:12.7]	10.6 [7.9:13.7]	0.9477
LAVI	33.0 [28.1:40.4]	41.5 [29.4:48.2]	31.5 [28.0:36.1]	0.0029
LVMI	102.4 [83.4:114.7]	105.9 [102.2:126.0]	97.4 [79.9:111.8]	0.0284
LVDD*	26 (34.7%)	12 (46.2%)	14 (28.6%)	0.2050

*BMI* body mass index, *SBP* systolic blood pressure, *DBP* diastolic blood pressure, *HDL* high density lipoprotein cholesterol, *RAAS* renin angiotensin aldosterone system modifying therapy, *AA* aldosterone antagonists, *ARB* aldosterone receptor blocker, *ACEI* angiotensin converting enzyme inhibitor, *Loop* loop diuretics, *BB* beta blocker, *CCB* calcium channel blocker, *EF* ejection fraction, *LAVI* left atrial volume index, *LVMI* left ventricular mass index, *LVDD* left ventricular diastolic dysfunction. *LAVI > 34 and e’ < 10

### Biomarker prediction of new onset HFpEF

Two time-point biomarker analysis of 5 disease associated biomarkers was conducted in patient samples prior to new onset HFpEF and is described in Table 2. The first of these was measured a median of 2.8 years and the second a median of 1.6 years prior to the diagnosis of new onset HFpEF. Accordingly, the median time between biomarker measurements was 1.2 years.

**Table 2:** Unadjusted biomarker concentrations at study baseline and follow up time points

Biomarker	HFpEF progressor	HFpEF Non progressor	*p*-value
BNP Baseline	107.7 [51.4:179.2]	39.8 [19.6:74.5]	< 0.001
BNP Follow up	117.0 [63.9:179.0]	47.2 [25.4:79.1]	< 0.001
Galecin-3 Baseline	15.9 [14.8:18.6]	14.9 [12.5:18.3]	0.0663
Galectin-3 Follow up	17.9 [14.8:20.0]	15.4 [12.5:18.0]	0.0090
hsTroponin I Baseline	7.7 [5.6:10.0]	5.8 [4.8:7.7]	0.0647
hsTroponin I Follow up	7.2 [5.8:10.9]	6.2 [4.5:7.6]	0.0202
sST2 Baseline	25.3 [21.3:30.5]	22.9 [20.5:29.0]	0.3711
sST2 Follow up	25.3 [22.6:29.8]	24.6 [19.6:32.3]	0.4488
IL6 Baseline	1.4 [1.0:2.1]	1.1 [0.76:1.8]	0.0731
IL6 Follow up	1.4 [1.0:1.8]	1.0 [0.79:1.9]	0.0861

*BNP* b-type natriuretic peptide, *hsTroponin I* high sensitivity troponin I, *sST2* soluble suppression of tumorigenicity 2, *IL6* interleukin 6. Units, BNP pg/mL; Galectin-3 ng/mL; hsTroponin I pg/mL; sST2 ng/mL; IL6 pg/mL

Logistic regression modelling was used to investigate the ability of the 5 studied biomarkers to predict the future development of new onset HFpEF (Table 3). Using both unadjusted and adjusted models (adjusted for all other biomarkers at corresponding time-point), BNP was a significant predictor of new onset HFpEF using data from both baseline and follow-up patient profiling (p ≤ 0.001), but BNP change over time was not predictive. Area under curve (AUC) for BNP at baseline, follow-up, and longitudinal change for new onset HFpEF was 0.74 (95% CI = [0.63, 0.86]), 0.81 (95% CI = [0.71, 0.90]), and 0.58 (95% CI = [0.45, 0.71], respectively.

**Table 3:** Predicting future new onset HFpEF with five biomarkers

	Unadj. OR [95% CI]	*p*	Adj. OR [95% CI]	*p*	AUC
Gal3 Baseline	1.08 [0.99,1.18]	ns	1.08 [0.97,1.21]	ns	0.62 [0.50,0.74]
Gal3 Follow-up	1.15 [1.03,1.28]	0.011	1.17 [1.02,1.34]	0.027	0.67 [0.55,0.78]
Gal3 delta	1.04 [0.90,1.22]	ns	1.32 [1.04,1.67]	0.024	0.63 [0.50,0.76]
sST2 Baseline	1.04 [0.98,1.09]	ns	1.06 [1.00,1.13]	ns	0.56 [0.43,0.69]
sST2 Follow-up	1.03 [0.99,1.08]	ns	1.03 [0.97,1.08]	ns	0.55 [0.43,0.67]
sST2 delta	1.03 [0.95,1.11]	ns	1.01 [0.93,1.10]	ns	0.51 [0.37,0.65]
IL6 Baseline	1.28 [0.91,1.80]	ns	1.28 [0.89,1.85]	ns	0.62 [0.50,0.74]
IL6 Follow-up	1.08 [0.78,1.49]	ns	1.11 [0.72,1.71]	ns	0.61 [0.49,0.73]
IL6 delta	0.81 [0.56,1.16]	ns	0.76 [0.51,1.14]	ns	0.54 [0.40,0.68]
hsTropI Baseline	1.19 [1.03,1.38]	0.017	1.21 [1.04,1.42]	0.017	0.62 [0.49,0.76]
hsTropI Follow-up	1.19 [1.03,1.37]	0.016	1.07 [0.93,1.22]	ns	0.65 [0.53,0.77]
hsTropI delta	1.14 [0.92,1.40]	ns	1.07 [0.85,1.35]	ns	0.58 [0.44,0.72]
ln(BNP) Baseline	2.85 [1.62,5.02]	< .001	2.71 [1.47,5.01]	0.001	0.74 [0.63,0.86]
ln(BNP) Follow-up	5.16 [2.34,11.4]	< .001	5.13 [2.14,12.3]	< .001	0.81 [0.71,0.90]
ln(BNP) delta	1.58 [0.79,3.16]	ns	1.70 [0.79,3.64]	ns	0.58 [0.45,0.71]
All markers model—Baseline	–	–	–	–	0.82 [0.73,0.92]
All markers model—Follow-up	–	–	–	–	0.86 [0.79,0.94]
All markers model—delta	–	–	–	–	0.68 [0.55,0.81]

*Adj. OR* Odds Ratio Adjusted for all other biomarkers at corresponding time-point, *Gal3* Galectin-3, *sST2* soluble suppression of tumorigenicity 2, *IL6* interleuckin 6, *hsTroponin * high sensitivity troponin I, *BNP* b-type natriuretic peptide

hsTroponin I at baseline and follow-up was a significant predictor of future new onset HFpEF in unadjusted models (*p* < 0.05) and remains significant after controlling for the other biomarkers at baseline only. Longitudinal change in hsTroponin I was not a significant predictor of disease onset. Galectin-3 was only predictive of new onset HFpEF at the time point closest to the event, and was significant in both unadjusted and adjusted models (*p* < 0.05). Change in Galectin-3 becomes a significant predictor of HFpEF only after controlling for levels of the other biomarkers (*p* < 0.05). AUC for Galectin-3 at follow-up for new onset HFpEF was 0.67 (95% CI = [0.55, 0.78]). Baseline, follow-up, and longitudinal change IL-6 and sST2 data were not predictive of future development of new onset HFpEF in this study. When conducting a repeat measure analysis using baseline and follow up data on the 5 biomarkers there were no significant findings observed (data not shown).

To investigate the relationship between biomarker levels at both study time points and time to heart failure diagnosis, Cox proportional hazard models were carried out. Only baseline IL-6 presented significance in the model (unadjusted model HR 1.437 [95% CI = 1.111, 1.859], *p* = 0.006; age/sex adjusted model HR 1.440 [95% CI = 1.101, 1.883], *p* = 0.007). Therefore, a one unit increase in baseline IL-6 is associated with a 44% increase in the rate of HFpEF.

### Echocardiography prediction of new onset HFpEF

Logistic regression modelling was also used to investigate the ability of echocardiographic parameters to predict the future development of new onset HFpEF. When adjusting for all other biomarkers at corresponding time-point, only LAVI was a significant predictor of new onset HFpEF using data from baseline (*p* < 0.05), follow-up (*p* ≤ 0.001), and change over time (*p* < 0.05). AUC for LAVI at baseline, follow-up, and longitudinal change for new onset HFpEF was 0.71 (95% CI = [0.57, 0.85]), 0.79 (95% CI = [0.67, 0.92]), and 0.68 (95% CI = [0.54, 0.83], respectively.

### Biomarker combination models and clinical relative risk matrix

To further refine risk prediction of new onset HFpEF, a multimarker analysis was conducted whereby a model was created using baseline biomarker data on BNP, hsTroponin I, and Galectin-3. The models were restricted to these variables since sST2, IL-6 and all delta variables (except adjusted Galectin-3) in logistic regression models were not significant predictors of later HFpEF development and were thus excluded from this analysis.

The results in Table 4 suggest that when the model was applied to the follow-up data it was more accurate at predicting future HFpEF compared to the baseline data. This was as expected, since the values are closer in time to the diagnosis of HFpEF. Net Reclassification Improvement (NRI) between models which include medications (RAAS-modifying therapies, diuretics and β-blockers) versus excluding medications indicate that it is not necessary to take account of patient medications at moderately high (80%) sensitivity. However, Integrated Discrimination Improvement (IDI) between models including and excluding medications indicate that it may be necessary to take account of medications at other sensitivity thresholds. Table 5 highlights BNP, hsTroponin I, and Galectin-3 individually in predicting future HFpEF using follow-up data only.

**Table 4:** ROC curve AUCs for three-biomarker model at two time-points

	Excluding Meds		Including Meds		IDI [95% CI]*	NRI [95% CI]*†
	AUC [95% CI]	Tjur R^2^	AUC [95% CI]	Tjur R^2^		
Baseline data	0.79 [0.69,0.88]	22.9%	0.80 [0.71,0.89]	28.4%	5.4 [0.3,10.4]	5.0 [− 13.3,23.3]
Follow-up data	0.85 [0.77,0.93]	30.0%	0.86 [0.78,0.93]	36.3%	6.2 [0.6,11.8]	11.8 [− 4.9,28.6]

**Table 5:** Predicting later heart failure with three biomarkers—follow-up data only

	Excluding Meds			Including Meds		
	Coef	SE	*p*	Coef	SE	*p*
Intercept	− 10.71	2.39	< .001	− 10.14	2.51	< .001
ln(BNP)	1.58	0.44	< .001	1.66	0.50	0.001
hsTroponin I	0.07	0.07	0.32	0.06	0.09	0.50
Galectin-3	0.16	0.07	0.017	0.14	0.08	0.06
RAAS	–	–	–	− 1.34	0.73	0.07
Diuretics	–	–	–	1.93	1.32	0.14
β-blockers	–	–	–	0.56	0.65	0.39

*BNP* b-type natriuretic peptide, *hsTroponin I* high sensitivity Troponin I, *RAAS* renin angiotensin aldosterone system modifying therapy

**Fig. 1:**
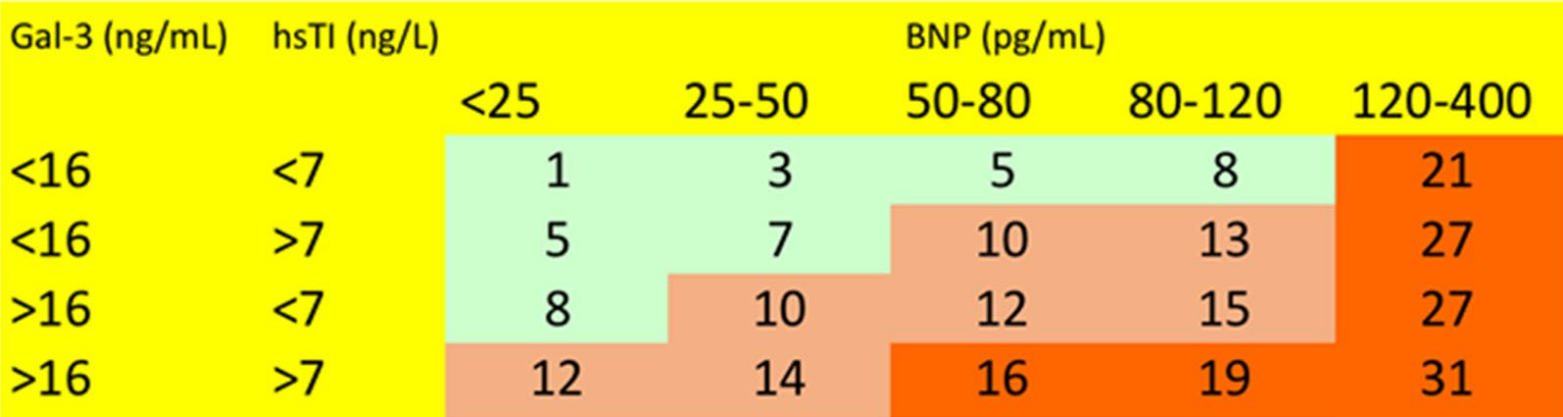
Novel relative risk model of new onset HFpEF development. Relative risk model for the development of new onset HFpEF stratified by quintiles of BNP and median hsTroponin I and Galectin-3 thresholds.

The relative risk matrix (Fig. 1) for new onset HFpEF was based on approximate quintiles of BNP levels and median biomarker thresholds of hsTroponin I and Galectin-3. We used quintiles of BNP and high/low combinations of the other biomarkers as point in time BNP was the strongest predictor of new onset HFpEF in all analyses. The lowest thresholds were defined as BNP (< 25 pg/mL), hsTroponin I (< 7 ng/L) and Galectin-3 (< 16 ng/mL) and patients in this category were allocated a relative risk of 1. Arbitrarily, red/green areas in the matrix are used to denote patients as either at high risk (> 20 fold the reference value) or low risk (< tenfold the reference value) respectively. Intermediate relative risks (10–20 fold) are denoted by orange areas in the matrix.

## Discussion

Heart failure with preserved ejection fraction (HFpEF) is a prevalent and debilitating form of heart failure and, unlike Heart failure with reduced ejection fraction (HFrEF), successive large scale randomised trials of RAAS modifying therapies have failed to provide evidence based, disease modifying therapies. This places focus on HFpEF prevention in the community, where HFpEF is the most prevalent form. HFpEF is characterised by progressive onset of cardiac remodelling and ventricular dysfunction, and the gradual natural progression of this disease provides us with the opportunistic window to apply biomarker-based risk stratification approaches. This will help to identify patients early on who could benefit from a more focused management regime that can attenuate disease progression, in a similar approach described in STOP-HF and PONTIAC studies [1, 19].

Our present study specifically uses circulating levels of five physiologically distinct biomarkers that reflect the pathologies at play in HFpEF – hsTroponin I, BNP, Galectin-3, sST2 and IL-6 – to show the potential of sequential biomarker analysis in identifying asymptomatic, event-free patients with risk factors for future HFpEF development. The objective was to investigate the utility of single time point analysis and longitudinal change in the concentrations of these biomarkers to identify those at highest risk and predict future new onset HFpEF. We establish that hsTroponin I and BNP at baseline and follow-up, as well as Galectin-3 at follow up, are independent predictors of future new onset HFpEF in an asymptomatic, event-free population. We then further present a multimarker model that can risk stratify patients for new onset HFpEF. With sequential evaluation of these and other biomarkers in larger at-risk populations, new risk stratification algorithms may favorably alter the growing HFpEF epidemic and efficiently guide intensive prevention.

The potential biomarker value of hsTroponin, BNP, and Galectin-3 in the context of risk-stratification and HFpEF diagnosis have been previously reported. Both hsTroponin and BNP have previously established/published thresholds for risk stratification. For example, it has been suggested that hsTroponinI < 4/ < 6 ng/L is indicative of low risk and > 10/ > 12 ng/L is indicative of at risk, in females/males respectively [20–22]. While a BNP risk predictor cutoff of 50 pg/mL has been indicated [23]. These cutoffs are similar to what we have identified in this study and are highlighted in Fig. 1. With regards to hsTroponin, this is a well-established marker of myocardial injury in acute coronary syndromes, but its role in HF is less well defined [24]. Prior studies [25, 26], including the latest PARAMOUNT trial [27] have shown that the majority of HFpEF patients had hsTroponin concentrations above the threshold for diagnosis of myocardial injury. This elevation is likely a result of increased left ventricular size and mass, increased left atrial size, collectively contributing to increased wall stress, in addition to myocardial hypertrophy and fibrosis [28], alterations in microcirculation all of which further contribute to relative ischemia [29]. Overall, this finding of elevated troponin in stable HFpEF is suggestive of ongoing subclinical myocardial injury that may explain the high morbidity and mortality associated with HFpEF [27, 30]. While our results show that single time point measurements of hsTroponin I is predictive of future new onset HFpEF in asymptomatic people, serial changes in plasma concentrations over time was not a significant predictor in this study population. hsTroponin has also been studied in other longitudinal community based cohorts (FRAMINGHAM, CHS, MESA, PREVEND), although these have been restricted to single time point analysis at study baseline, with much longer follow periods to time of incident new onset HFpEF, approximately 12 years after baseline biomarker analysis [31]. Combining the data from these four cohorts, de Boer et. al. conducted a multivariable-adjusted pooled analysis and identified hsTroponin to be a significant predictor of future incident HFpEF [31].

BNP, a peptide hormone released primarily from the cardiac ventricles in response to myocyte stretch or injury, is currently the most widely used biomarker to aid diagnosis of HF or to risk stratify those at risk of developing HF [32]. Notably, the utility of BNP can be further enhanced by incorporating it into a risk model to predict outcome and stratify HFpEF patients on an individual patient basis [33], allowing for tailored care and targeted management of these patients. There are few studies on the clinical utility of serial BNP testing in HFpEF patients, let alone in ambulatory patients and specifically for HFpEF. Hence, we set forth to determine if serial changes in BNP provides clinically important prognostic information in predicting future new onset HFpEF in asymptomatic patients. Maisel et. al has previously shown that changes in BNP overtime in parallel with weight gain are associated with decompensation in HFpEF patients [34], however it appears this trend does not carry over to a non-HF population. In our study, changes in BNP over time was not predictive of future HFpEF approximately 1.6 years later, and perhaps the true value of serial BNP testing to identify risk is of greater benefit in established HF populations. Interestingly, however, single time point analysis of BNP in our asymptomatic cohort with cardiovascular risk factors was sufficient to identify future HFpEF risk. Similar observations have been made in other, larger community-based cohorts (FRAMINGHAM, CHS, MESA, PREVEND) whereby single time point natriuretic peptide analysis is predictive of future new onset HFpEF [31].

Galectin-3, with its well documented roles in the promotion of cardiac fibrosis, has been shown to be predictive of cardiovascular mortality and HF related hospitalization events in both acute and chronic HF, all independent of BNP [11, 35–41]. Keeping in mind the unique differences of Galectin-3 versus BNP, and the predictive power of Galectin-3 in patients who have been previously diagnosed with HF, Galectin-3 is postulated to have great potential in being able to predict left ventricular dysfunction and cardiovascular events in asymptomatic patients who are at higher risk of evolution to HF, with the notion that active fibrosis may precede clinical manifestations of HF by many years. While previous studies have examined the prognostic value of Galectin-3 in patients with chronic HFpEF and HFrEF (34) [39], the added prognostic value of the serial measurements of Galectin-3 is less well established. Although some prior studies have had conflicting results regarding the independent value of Galectin-3 [38, 40, 42], Motiwala et. al. have demonstrated that the addition of a second measurement of Galectin-3 at 6 months to the baseline value provided significantly greater prognostic information in HFrEF patients [43]. In fact, significant categorical changes in Galectin-3 level according to the threshold of 20.0 ng/mL were predictive of cardiovascular events, with an increase from below to above 20.0 ng/mL conferring an increased risk, and a decrease in this level was associated with fewer cardiovascular events. Similarly, an increase in the level by ≥ 15% at any 3-month follow-up interval conferred worse prognosis in this HFrEF cohort, even after extensive clinical adjustment. This was consistent with the data from HFrEF patients as described by van der Velde et al. 44. Finally, not distinguishing between HFrEF and HFpEF, Ho et al. found that higher circulating Galectin-3 concentrations are associated with increased risk for new-onset HF and all-cause mortality in the community [45]. Thus far, our study is the only HFpEF focused study that has looked at the ability of Galectin-3 over two time points to predict future heart failure development in an asymptomatic population. We demonstrated that while galectin-3 was only a significant predictor at follow-up, this analysis was still 1.6 years before the HFpEF event. Looking at HFpEF development as a continuum, a pathophysiological circulating Galectin-3 signal may appear much closer to the event compared with BNP and hsTroponin I as it is likely that myocyte stress signals will appear before fibrosis related signals. This may be the reason why Galectin-3 was not predictive of future HFpEF development in the combined community cohort analysis by de Boer et. al. as the point in time Galectin-3 baseline measurement was on average 12 years before heart failure diagnosis [31]. In our study, the data also highlights that change in Galectin-3 levels over time in the model adjusted for all other biomarkers was also a significant predictor of new onset HFpEF, indicating the potential clinical value in serial Galectin-3 measurements in patients that have been previously signified as high-risk.

To further enhance our risk prediction for the development of HFpEF, we then proceeded to use the incremental value of our biomarkers of interest that reflect the different pathological processes operative in HFpEF. Using an unadjusted biomarker combination of hsTroponin I, BNP, and Galectin-3, our study could significantly predict future HFpEF using both baseline (AUC 0.77 [0.68,0.87]) and follow-up data (AUC 0.86 [0.79,0.94]). At 80% sensitivity, this predictive model is independent of having to account for medications taken by patients. Based on these findings, the baseline time point of approximately 2.8 years prior to HFpEF onset, we demonstrate that BNP and hsTroponin I are able to predict risk of later HFpEF development. At the follow-up time point analysis, which equates to approximately 1.6 years before HFpEF onset, BNP and Galectin-3 are able to predict HFpEF risk. In adjusted models for all other biomarkers, change in Galectin-3 levels over time was a significant predictor of new onset HFpEF. In logistic regression models, sST2 and IL-6 were unable to predict the future development of HFpEF in our study population. When examining the echocardiography data, only LAVI was a significant predictor of future new onset HFpEF when adjusted for all other biomarkers. Although this was significant at baseline, follow-up and change over time, biomarker combination models were more predictive. This is reassuring as the application of biomarker profiling of large populations of asymptomatic, event-free patients with risk factors for future HFpEF development is more attractive than echocardiography assessment, from a resource and logistical perspective.

The approach of using clinical relative risk matrices to aid clinical decision making has been established for several decades, with the Framingham Risk Score or ESC SCORE risk as notable examples. As with the clinical relative risk matrix presented here, there are a number of important limitations to consider: first, the relative risk matrix calculates the risk of new onset HFpEF, but not other cardiovascular or other health conditions; second, the risk matrix is correct only for the population from which it derives and could easily over or under estimate the relative risk on other populations; third, the biomarker cutoffs and definition of high and low relative risks are arbitrary and must be considered in the context of the population and the objectives of risk prediction. As heart failure development is time-varying, more work is needed to validate the predictive value of these biomarkers for future new onset HFpEF over a greater follow-up period or across multiple timepoints. In addition, external validation of the relative risk matrix to adapt the algorithm to other populations and other outcomes before evaluating the usefulness of this approach clinically.

## Conclusions

In conclusion, our study is the first to demonstrate that in an asymptomatic, event-free community base cohort, initial high-risk for future new onset HFpEF in the population could be identified through risk profiling with BNP and hsTroponin. Once high-risk was established, the patient could be monitored longitudinally with the aid of serial Galectin-3 measurements. As depicted in Fig. 1, a novel relative risk matrix was created as an example of how this might be used clinically in attempts to support the risk prediction of new onset HFpEF. Such a proposed model would need to be validated in independent and larger patient cohorts to confirm the clinical value of such a risk predictor. In addition, re-assessment of low risk graduating to high risk is less clear at present but further work from longitudinal community cohorts such as STOP-HF and others will help determine this.

## Limitations

The present study is hindered by a few unavoidable limitations. The number of patients in the study population was relatively small and was carried out in a single region within the Irish health care system. Therefore, the results may differ in populations with different levels of absolute risk and ethnicities and this may not be generalizable to other health care settings. However, this justifies the need for validation of our biomarker combination models and the relative risk matrix in an independent population.

## Data Availability

The datasets generated and/or analysed during the current study are not publicly available but are available from the corresponding author on reasonable request.
